# Modeling the disturbing effect on the aist small spacecraft based on the measurements data

**DOI:** 10.1038/s41598-022-05367-9

**Published:** 2022-01-25

**Authors:** A. V. Sedelnikov, V. V. Salmin

**Affiliations:** Department of Space Engineering, Samara National Research University, Moscow Shosse, 34, Samara, 443086 Russia

**Keywords:** Engineering, Mathematics and computing

## Abstract

The paper considers the issues of reconstruction of the power disturbing effect on the prototype of the small spacecraft "Aist". The measurements data of the components of the induction vector of the Earth's magnetic field by means of two three-component magnetometers were used for the reconstruction. The obtained results can be used to assess microaccelerations in the internal environment of small spacecraft, as well as to study their uncontrolled rotational motion.

## Introduction

**Figure 1:**
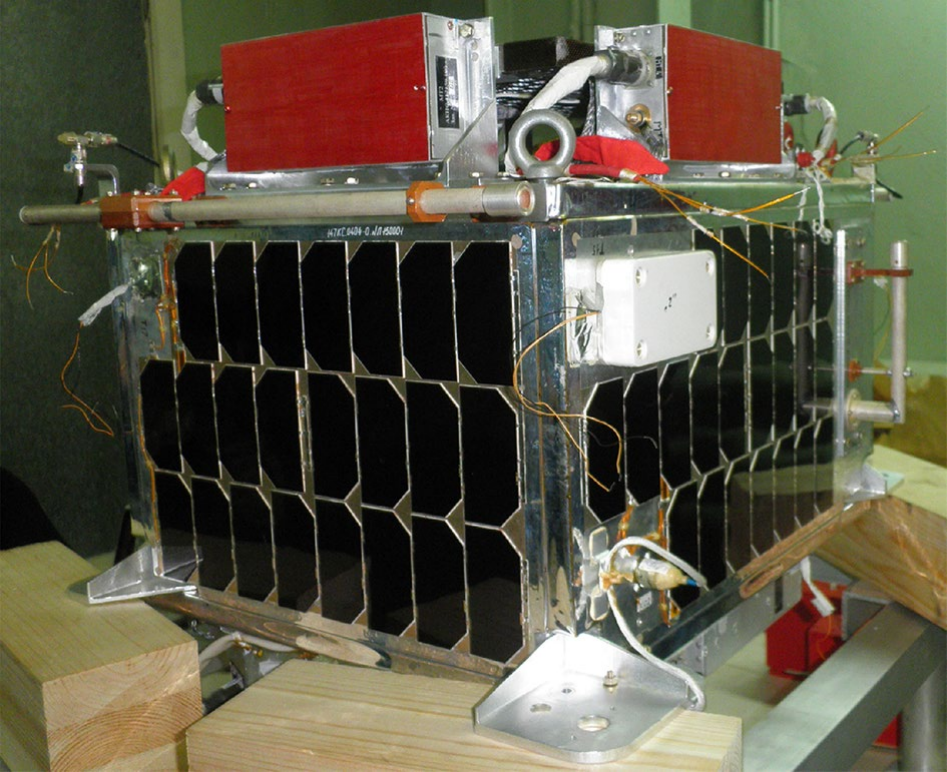
There is the small spacecraft prototype "Aist. (cited in^4^).

The small spacecraft have been widely used relatively recently. However, they have been actively developing lately due to their competitive advantages^1,2^. At the same time, the features of the orbital motion of small spacecraft have been insufficiently studied. Thus, attempts to reduce the angular speed of rotation of the small spacecraft prototype "Aist" (Fig. 1) using magnetic actuators did not lead to the desired result^3^.

**Figure 2:**
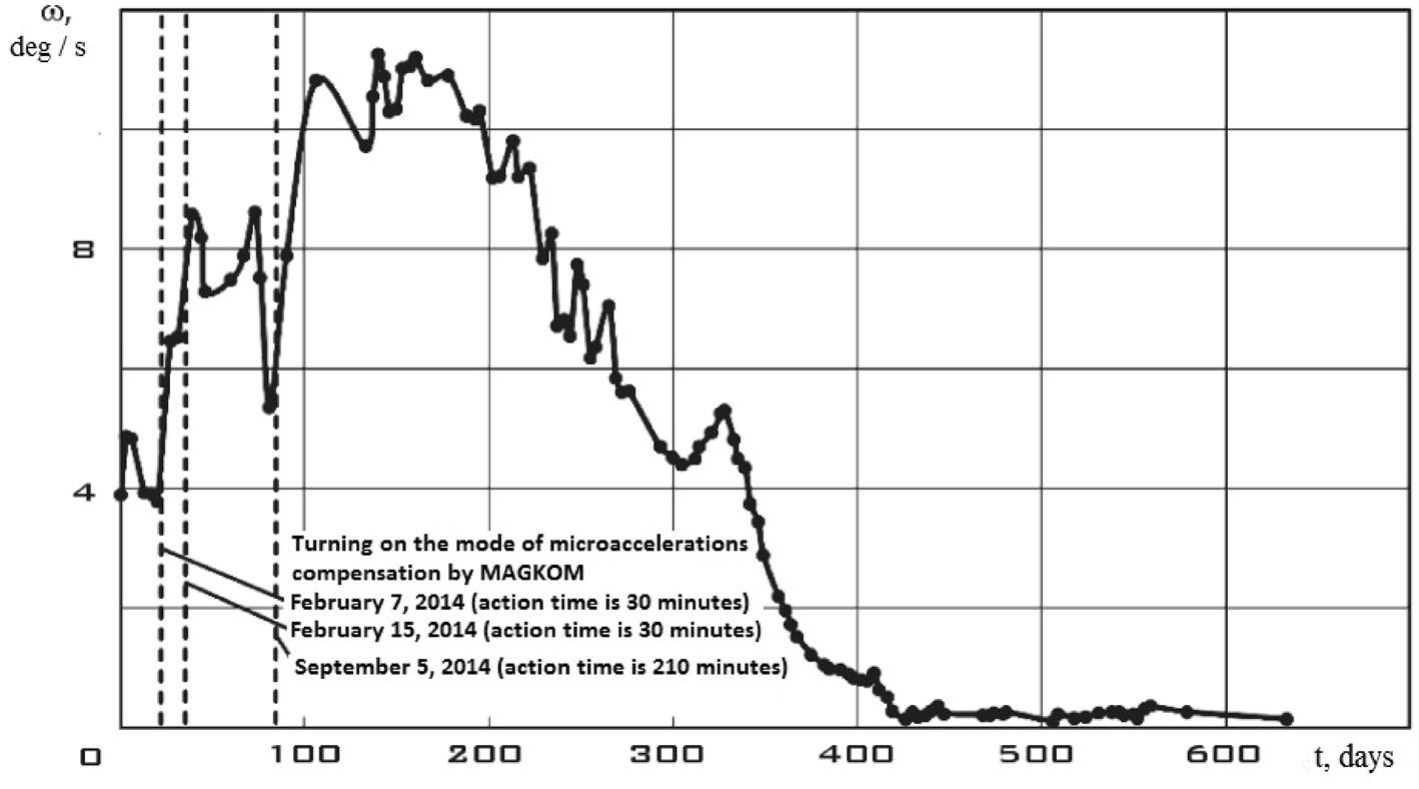
There is dynamics of changes in the angular velocity modulus of the small spacecraft prototype "Aist" rotation (dotted lines indicate attempts to reduce the angular velocity by magnetic actuators) (cited in^5^).

The small spacecraft independently stabilized its rotation relative to the center of mass without control actions after some time (Fig. 2.^5^). The work^5^ investigated the stability of the rotational motion of the small spacecraft prototype "Aist" relative to the components of the angular velocity vector. The motion will be unstable with respect to the angular coordinates since all three angular coordinates are cyclic.

The stabilization time is quite long as can be seen from Fig. 2. It is commensurate with the active life of the smallest spacecraft (3 years). Therefore, a more detailed study of the disturbing force effect on a small spacecraft is necessary in order to develop effective control laws that significantly reduce the stabilization time. In this sense, the choice of the small spacecraft prototype "Aist" for modeling such an impact is very successful, since during separation it received a significant initial angular velocity^6^.

Thus, the problem posed in this work is important and relevant for effective control of the movement of a small spacecraft relative to the center of mass. High values of the angular velocity reduce the quality of the transmitted telemetry information, the efficiency of the magnetic actuators, and also impede the successful implementation of gravitational-sensitive processes on board a small spacecraft^5^.

## Mathematical model of the evolution of the small spacecraft prototype "Aist" around the center of mass

**Figure 3:**
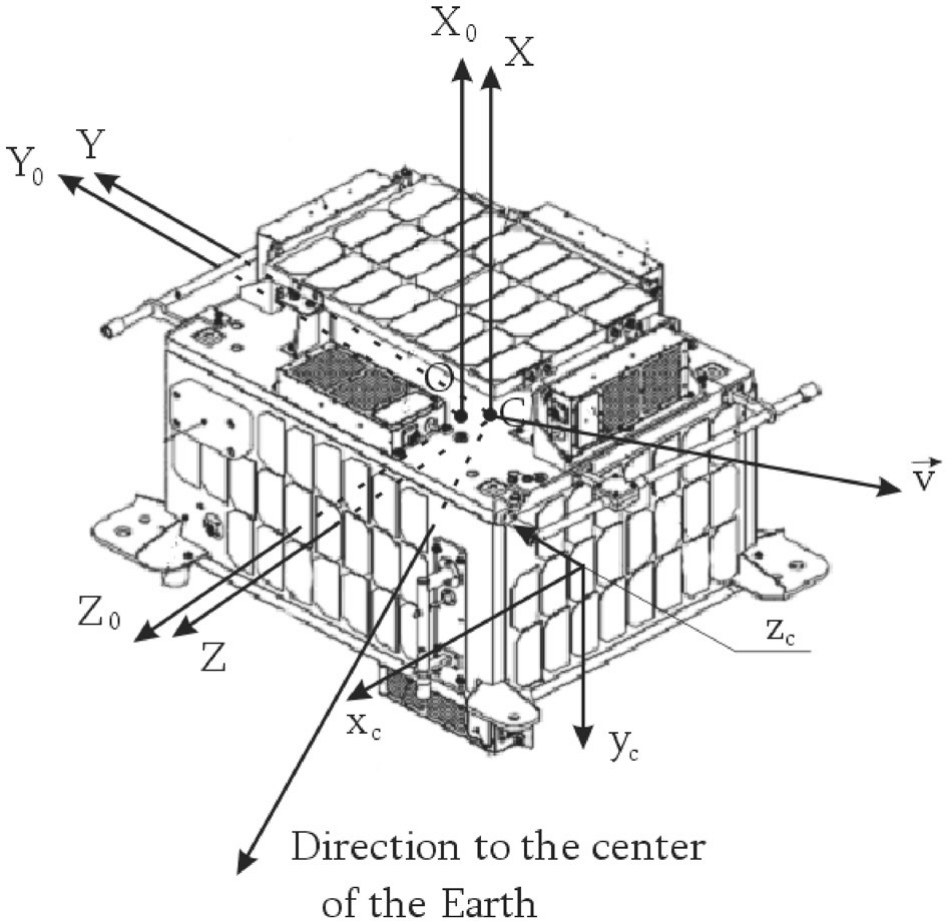
There are used coordinate systems.

We use the dynamic Euler equations in a coordinate system whose axes are parallel to the main axes of inertia, and the center coincides with the center of mass to simulate the rotational motion of the small spacecraft prototype "Aist", (CXYZ):

1 $$\left\{ \begin{gathered} I_{xx} \dot{\omega }_{x} - I_{xy} \dot{\omega }_{y} - I_{xz} \dot{\omega }_{z} + \omega_{y} \left( {I_{zz} \omega_{z} - I_{xz} \omega_{x} - I_{yz} \omega_{y} } \right) - \omega_{z} \left( {I_{yy} \omega_{y} - I_{xy} \omega_{x} - I_{yz} \omega_{z} } \right) = M_{x} \hfill \\ \,I_{yy} \dot{\omega }_{y} - I_{xy} \dot{\omega }_{x} - I_{yz} \dot{\omega }_{z} + \omega_{z} \left( {I_{xx} \omega_{x} - I_{xy} \omega_{y} - I_{xz} \omega_{z} } \right) - \omega_{x} \left( {I_{zz} \omega_{z} - I_{xz} \omega_{x} - I_{yz} \omega_{y} } \right) = M_{y} \, \hfill \\ I_{zz} \dot{\omega }_{z} - I_{xz} \dot{\omega }_{x} - I_{yz} \dot{\omega }_{y} + \omega_{x} \left( {I_{yy} \omega_{y} - I_{xy} \omega_{x} - I_{yz} \omega_{z} } \right) - \omega_{y} \left( {I_{xx} \omega_{x} - I_{xy} \omega_{y} - I_{xz} \omega_{z} } \right) = M_{z} \hfill \\ \end{gathered} \right.,$$

where $$\hat{I} = \left[ \begin{gathered} \,\,\,I_{xx} \,\,\, - I_{xy} \,\,\, - I_{xz} \hfill \\ - I_{xy} \,\,\,\,\,I_{yy} \,\,\, - I_{yz} \hfill \\ - I_{xz} \,\, - I_{yz} \,\,\,\,\,I_{zz} \hfill \\ \end{gathered} \right]$$ is inertia tensor of a small spacecraft in a coordinate system CXYZ (Fig. 3); $\vec{\varepsilon }\left( {\dot{\omega }_{x} ,\dot{\omega }_{y} ,\dot{\omega }_{z} } \right)$ and $\mathop{\omega }\limits^{\rightharpoonup} \left( {\omega_{x} ,\omega_{y} ,\omega_{z} } \right)$ are respectively vectors of angular acceleration and angular velocity of rotation of the small spacecraft prototype "Aist" around the center of mass in the coordinate system CXYZ; $\vec{M}\left( {M_{x} ,M_{y} ,M_{z} } \right)$ is vector of the external perturbing moment acting on a small spacecraft.

*OX*_0_*Y*_0_*Z*_0_ is main bound coordinate system; *CXYZ* is bound coordinate system, the axes of which are parallel to the axes of the main bound coordinate system, and the origin is at the center of mass (point C) of the small spacecraft; *x*_*c*_*y*_*c*_*z*_*c*_ is construction coordinate system of magnetometer No. 2 (this coordinate system is left-handed).

Let us assess the significance of external disturbances for the considered small spacecraft. The vector of the external disturbing moment can be represented as follows for real operating conditions of the small spacecraft prototype "Aist":

2 $$\vec{M} = \vec{M}_{aer} + \vec{M}_{grav} + \vec{M}_{mag} + \vec{M}_{cont} + \vec{M}_{{{\text{other}}}} ,$$

where $\vec{M}_{aer}$ is aerodynamic disturbance moment; $\vec{M}_{grav}$ is gravitational disturbance moment; $\vec{M}_{mag}$ is magnetic disturbance moment; $\vec{M}_{cont}$ is control moment, $\vec{M}_{{{\text{other}}}}$ – other disturbance moments not taken into account in the previous summands^7^.

The fourth term on the right-hand side of expression (2) is absent since the stabilization of the small spacecraft prototype "Aist" without control is considered. We consider the gravitational disturbing moment, estimating its value by the formula^8^:

3 $$\left| {\vec{M}_{grav} } \right| = \left| {\frac{3\,\mu }{{r^{3} }}\vec{e}_{r} \times \left( {\hat{I} \cdot \vec{e}_{r} } \right)} \right|,$$

where $\mu$ is gravitational parameter of the Earth; $r$ is orbital radius of the small spacecraft prototype "Aist; $\vec{e}_{r}$ is unit vector of direction to the center of the Earth (Fig. 3).

When $r = 6871\,\,km$ and $$\hat{I} = \left( \begin{gathered} \,\,\,1,7\,\,\,\, - 1,0\,\,\,\,\, - 0,8 \hfill \\ - 1,0\,\,\,\,\,\,\,1,2\,\,\,\,\,\, - 1,1 \hfill \\ - 0,8\,\,\,\, - 1,1\,\,\,\,\,\,\,\,\,1,5 \hfill \\ \end{gathered} \right)$$ according to (3) we have:

4 $$\begin{gathered} \left| {\vec{M}_{grav} } \right| = 3,686 \cdot 10^{ - 6} N \cdot m \cdot \max \left( {\left| {\left( \begin{gathered} 1 \hfill \\ 0 \hfill \\ 0 \hfill \\ \end{gathered} \right) \times \left( {\left( \begin{gathered} \,\,\,1,7\,\,\,\, - 1,0\,\,\,\,\, - 0,8 \hfill \\ - 1,0\,\,\,\,\,\,\,1,2\,\,\,\,\,\, - 1,1 \hfill \\ - 0,8\,\,\,\, - 1,1\,\,\,\,\,\,\,\,\,1,5 \hfill \\ \end{gathered} \right) \cdot \left( \begin{gathered} 1 \hfill \\ 0 \hfill \\ 0 \hfill \\ \end{gathered} \right)} \right)} \right|;\,\,\left| {\left( \begin{gathered} 0 \hfill \\ 1 \hfill \\ 0 \hfill \\ \end{gathered} \right) \times \left( {\left( \begin{gathered} \,\,\,1,7\,\,\,\, - 1,0\,\,\,\,\, - 0,8 \hfill \\ - 1,0\,\,\,\,\,\,\,1,2\,\,\,\,\,\, - 1,1 \hfill \\ - 0,8\,\,\,\, - 1,1\,\,\,\,\,\,\,\,\,1,5 \hfill \\ \end{gathered} \right) \cdot \left( \begin{gathered} 0 \hfill \\ 1 \hfill \\ 0 \hfill \\ \end{gathered} \right)} \right)} \right|;\,} \right. \hfill \\ \left. {\left| {\left( \begin{gathered} 0 \hfill \\ 0 \hfill \\ 1 \hfill \\ \end{gathered} \right) \times \left( \begin{gathered} \,\,\,1,7\,\,\,\, - 1,0\,\,\,\,\, - 0,8 \hfill \\ - 1,0\,\,\,\,\,\,\,1,2\,\,\,\,\,\, - 1,1 \hfill \\ - 0,8\,\,\,\, - 1,1\,\,\,\,\,\,\,\,\,1,5 \hfill \\ \end{gathered} \right) \cdot \left( \begin{gathered} 0 \hfill \\ 0 \hfill \\ 1 \hfill \\ \end{gathered} \right)} \right|} \right) = 3,686 \cdot 10^{ - 6} N \cdot m \cdot \max \left( {\left| {\left( \begin{gathered} 1 \hfill \\ 0 \hfill \\ 0 \hfill \\ \end{gathered} \right) \times \left( \begin{gathered} \,\,\,1,7 \hfill \\ - 1,0 \hfill \\ - 0,8 \hfill \\ \end{gathered} \right)} \right|;\,\,\left| {\left( \begin{gathered} 0 \hfill \\ 1 \hfill \\ 0 \hfill \\ \end{gathered} \right) \times \left( \begin{gathered} - 1,0 \hfill \\ \,\,\,1,2 \hfill \\ - \,1,1 \hfill \\ \end{gathered} \right)} \right|;\,\,\left| {\left( \begin{gathered} 0 \hfill \\ 0 \hfill \\ 1 \hfill \\ \end{gathered} \right) \times \left( \begin{gathered} - 0,8 \hfill \\ \, - 1,1 \hfill \\ \,\,\,\,1,5 \hfill \\ \end{gathered} \right)} \right|} \right) = \hfill \\ = 3,686 \cdot 10^{ - 6} \cdot \max \left( {\left| {0,8\vec{j} - \vec{k}} \right|} \right.;\,\left| { - 1,1\vec{i} + \vec{k}} \right|;\,\,\left. {\left| {1,1\vec{i} - 0,8\vec{j}} \right|} \right) \approx 5,5 \cdot 10^{ - 6} \,\,N \cdot m\, \hfill \\ \end{gathered}$$

Let us estimate the influence of the aerodynamic moment for the small spacecraft prototype "Aist" from the following data. The orbital radius of the small spacecraft has decreased by 5 km in three years. We believe that this happened solely due to the effect of the force of aerodynamic drag. This makes the estimate somewhat overestimated, since in reality other disturbing factors (light pressure, magnetic disturbance, micrometeorites, etc.) will also lead to deceleration. Let us take into account in the theorem on the change in kinetic energy the work of the forces of gravity and aerodynamic resistance:

5 $$\Delta T = A\left( {\vec{G}} \right) + A\left( {\vec{F}_{aer} } \right).$$

When $m_{spacecraft} = 39\,\,kg$, $R_{1} = 6865\,\,km$, $R_{0} = 6871\,\,km$ and $g \approx 8,44\,\,m/s^{2}$ we get:

$$A\left( {\vec{G}} \right) = m_{spacecraft} g\left( {R_{0} - R_{1} } \right) \approx 1,65\,\,MJ$$

We get it considering both orbits circular:

$$v_{1} = \sqrt {fM/R_{1} } \approx 7632,7\,\,m/s;\,\,\,v_{0} = \sqrt {fM/R_{0} } \approx 7629,9\,\,m/s$$

where *f* is gravitational constant, and *M* is mass of the Earth.

Then we have:

$$\Delta T = \frac{{m_{spacecraft} }}{2}\left( {v_{1}^{2} - v_{0}^{2} } \right) \approx 0,83\,\,MJ$$

We substitute the found estimates of changes in kinetic energy and work of gravitational forces into expression (5):

$$A\left( {\vec{F}_{aer} } \right) \approx - 0,82\,\,MJ$$

Let us estimate the distance covered by the small spacecraft prototype "Aist" in three years of flight:

$$s = 2\pi \overline{R}N$$

where $\overline{R} = \frac{6871 + 6865}{2} = 6868\,\,km$ is average orbital radius of a small spacecraft over three years; $N = \frac{3 \cdot 365,25 \cdot 24 \cdot 3600}{{5760}} \approx 16500$ is number of turns around the Earth in three years ($T = 5760\,\,s$ is average orbital period of a small spacecraft around the Earth).

Then we get:

$$s = 2\pi \overline{R}N = 2\pi \cdot 6868 \cdot 10^{3} \cdot 16500 \approx 7 \cdot 10^{11} m$$

We have an estimate of the modulus of the average aerodynamic force:

6 $$\overline{F}_{aer} = \frac{{A\left( {\vec{F}_{aer} } \right)}}{s} \approx \frac{{0,82 \cdot 10^{6} }}{{7 \cdot 10^{11} }} \approx 1,2 \cdot 10^{ - 6} \,N$$

The aerodynamic moment will not exceed with real values of the deviation of the center of pressure of the small spacecraft prototype "Aist" from the center of mass $5 \cdot 10^{ - 8} \,\,N \cdot m$.

Let us estimate the magnetic moment of the current of the entire small spacecraft considering the area of the circuit with the current to be its maximum overall dimensions $S = 0,5 \cdot 0,25 = 0,125\,\,m^{2}$. In this case, we have:

$$\left| {\vec{p}} \right| = i\,S$$.

**Figure 4:**
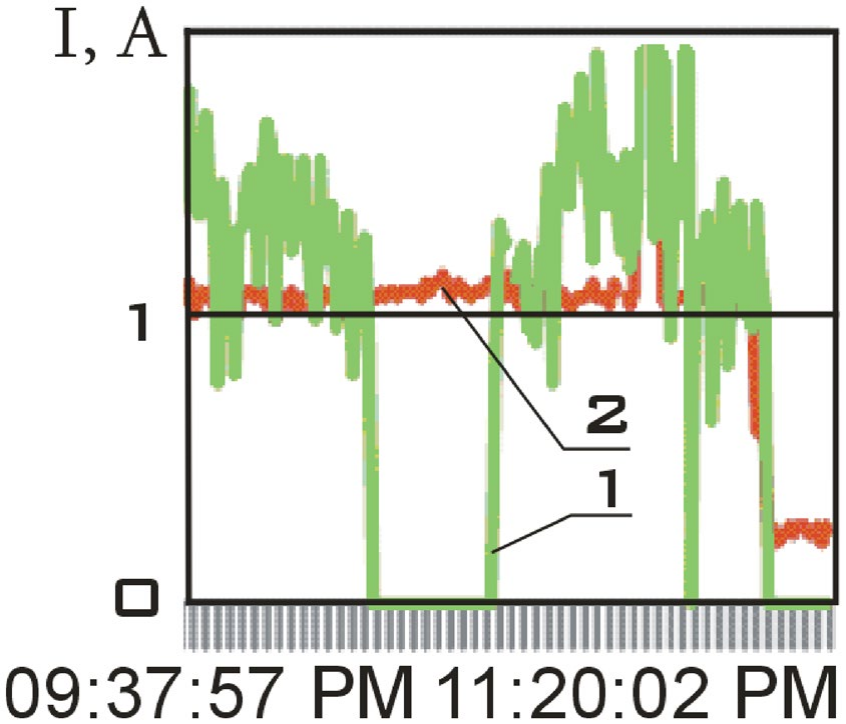
There is dynamics of currents in the power supply system of the small spacecraft prototype "Aist" according to telemetry data from 03/25/2014: 1 is battery charging current; 2 is current of power consumption of scientific equipment.

The solar battery panels, perpendicular to the *OX*_0_ axis of the main linked coordinate system, were structurally divided into two separate sections in the small spacecraft prototype "Aist". It is necessary to take into account the separate circuits with the current of the scientific and supporting equipment when evaluating the magnetic disturbing moment. The total charging current of the storage battery and the power consumption current of scientific equipment was recorded and was included in the telemetry information (Fig. 4).

Zero values of the battery charging current correspond to the shadow section of the small spacecraft orbit (Fig. 4).

Then you can get the maximum estimate of the magnetic moment acting on the small spacecraft prototype "Aist"^9^:

7 $$\vec{M}_{mag} = \sum\limits_{j = 1}^{n} {\vec{p}_{j} \times \vec{B}} \Rightarrow \left| {\vec{M}_{mag} } \right|_{\max } = \left| {\sum\limits_{j = 1}^{n} {\vec{p}} } \right| \cdot \left| {\vec{B}} \right| = 1,5 \cdot 10^{ - 5} \,N \cdot m$$

Thus, estimates (4), (6) and (7) show that the magnetic disturbing moment acting on the small spacecraft prototype "Aist" is dominant. Therefore, only it can be taken into account for the reconstruction of the disturbing effect in the first approximation. The presented estimates are in good agreement with similar estimates made for other spacecraft (Yamal-200, Yamal-201, Yamal-202^9^, Microsat^9^, Yamal-100, Egyptsat "^10^," Auriga "^11^).

Let us estimate the caused microaccelerations under the assumption that the angular velocity and angular acceleration are small quantities of the same order of magnitude. In this case, system (1) will have the form:

8 $$\left\{ \begin{gathered} I_{xx} \dot{\omega }_{x} - I_{xy} \dot{\omega }_{y} - I_{xz} \dot{\omega }_{z} = M_{x} \hfill \\ \,I_{yy} \dot{\omega }_{y} - I_{xy} \dot{\omega }_{x} - I_{yz} \dot{\omega }_{z} = M_{y} \, \hfill \\ I_{zz} \dot{\omega }_{z} - I_{xz} \dot{\omega }_{x} - I_{yz} \dot{\omega }_{y} = M_{z} \hfill \\ \end{gathered} \right.$$

since the squares of the angular velocities will have a higher order of smallness than the angular accelerations. Then, we obtain in the coordinate system *CXYZ* taking into account the values of the elements of the tensor of inertia:

9 $$\left\{ \begin{gathered} \dot{\omega }_{x} \approx 0,06\,M_{x} - 0,43\,M_{y} - 0,54\,M_{z} \hfill \\ \dot{\omega }_{y} \approx 0,29M_{x} + 0,78\,M_{y} - 0,33\,M_{z} \hfill \\ \dot{\omega }_{z} \approx 0,26\,M_{x} + 0,33\,M_{y} + 0,13\,M_{z} \hfill \\ \end{gathered} \right.$$

We consider that the module of microaccelerations in this case will be equal to:

10 $$\left| {\vec{w}} \right| = \sqrt {\dot{\omega }_{x}^{2} x^{2} + \dot{\omega }_{y}^{2} y^{2} + \dot{\omega }_{z}^{2} z^{2} }$$

(where x, y и z are the coordinates of the location point of the technological equipment in the *CXYZ* coordinate system). Equation (9) makes it possible to estimate the maximum values of microaccelerations from the previously considered disturbing factors. These estimates are shown in Table 1. The distance to the center of mass was taken equal to 0.3 m.

**Table 1:** There are maximum values of microaccelerations from the estimated disturbing factors.

The disturbing factor	$M_{grav}$	$F_{aer}$	$M_{aer}$	$M_{mag}$	$M_{cont}$
Microacceleration, *μm*/*s*^2^	1,50	0,03	0,01	5,00	80,00

Where $M_{grav}$ is gravitational disturbance moment; $F_{aer}$ is aerodynamic disturbance force; $M_{aer}$ is aerodynamic disturbance moment;$M_{mag}$ is magnetic disturbance moment; $M_{cont}$ is control torque of magnetic actuators.

There are maximum values of microaccelerations from the estimated disturbing factors.

The significance of the angular velocity will increase the estimates obtained. It will be necessary to take into account the normal accelerations since, in addition to tangential accelerations, in the formula (10). However, microaccelerations from magnetic disturbances will be significant for the successful implementation of gravitational-sensitive processes as shown by a number of studies^12–14^.

## Estimation of the angular velocity of the small spacecraft prototype "Aist" according to the measurements of the induction vector of the Earth's magnetic field

**Figure 5:**
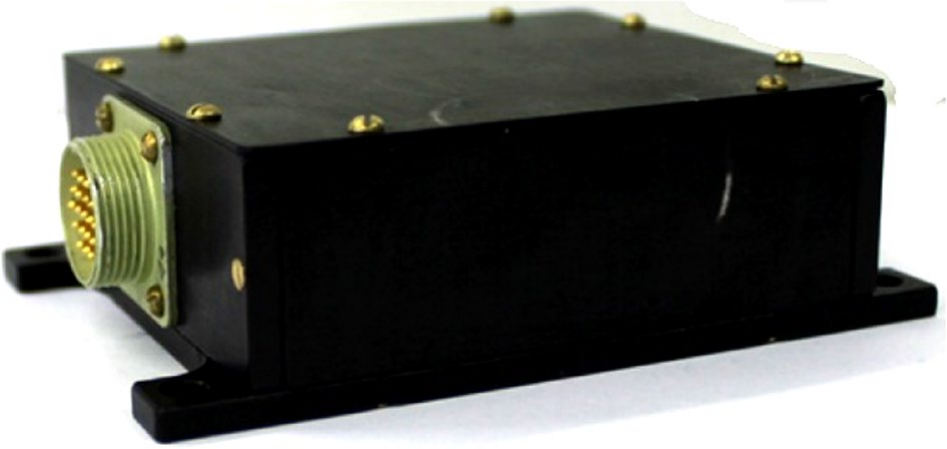
There is one of two magnetometers installed on the small spacecraft prototype "Aist". (cited in^4^).

We use the measurement data of the Earth's magnetic field induction vector using three-component magnetometers of the MAGKOM scientific equipment to estimate the angular velocity^6^ (Fig. 5).

The orientation of the induction vector of the Earth's magnetic field in the building axes of the magnetometers changes when the spacecraft rotates. It is the basis of the method for estimating the angular velocity of rotation using the well-known Boer formula^15,16^:

11 $$\vec{\omega } = \frac{{\vec{B} \times \left( {\dot{\vec{B}} - \frac{{\tilde{d}\vec{B}}}{d\,t}} \right)}}{{\vec{B}^{2} }}$$

The high frequency of measurements of the components of the induction vector makes it possible to neglect the first term in the parenthesis:

12 $$\vec{\omega } = - \frac{{\vec{B} \times \frac{{\tilde{d}\vec{B}}}{d\,t}}}{{\vec{B}^{2} }}$$

Formula (12) is convenient in that it makes it possible to estimate the angular velocity only on the basis of measurements of the components of the induction vector:

13 $$\begin{gathered} \omega_{{x_{1} }} = \left( {\frac{{B_{{y_{i} }} - B_{{y_{i - 1} }} }}{{\Delta \,t_{i} }}B_{{z_{i} }} - \frac{{B_{{z_{i} }} - B_{{z_{i - 1} }} }}{{\Delta \,t_{i} }}B_{{y_{i} }} } \right) \cdot \frac{1}{{B_{{x_{1} }}^{2} + B_{{y_{1} }}^{2} + B_{{z_{1} }}^{2} }}; \hfill \\ \omega_{{y_{1} }} = \left( {\frac{{B_{{z_{i} }} - B_{{z_{i - 1} }} }}{{\Delta \,t_{i} }}B_{{x_{i} }} - \frac{{B_{{x_{i} }} - B_{{x_{i - 1} }} }}{{\Delta \,t_{i} }}B_{{z_{i} }} } \right) \cdot \frac{1}{{B_{{x_{1} }}^{2} + B_{{y_{1} }}^{2} + B_{{z_{1} }}^{2} }}; \hfill \\ \omega_{{z_{1} }} = \left( {\frac{{B_{{x_{i} }} - B_{{x_{i - 1} }} }}{{\Delta \,t_{i} }}B_{{y_{i} }} - \frac{{B_{{y_{i} }} - B_{{y_{i - 1} }} }}{{\Delta \,t_{i} }}B_{{x_{i} }} } \right) \cdot \frac{1}{{B_{{x_{1} }}^{2} + B_{{y_{1} }}^{2} + B_{{z_{1} }}^{2} }}. \hfill \\ \end{gathered}$$

where $\vec{B}_{i} \left( {B_{{x_{i} }} ,B_{{y_{i} }} ,B_{{z_{i} }} } \right)$ and $\vec{B}_{i - 1} \left( {B_{{x_{i - 1} }} ,B_{{y_{i - 1} }} ,B_{{z_{i - 1} }} } \right)$ are measured respectively at times $t_{i}$ and $t_{i - 1}$ vectors of induction of the Earth's magnetic field, and $\Delta \,t_{i} = t_{i} - t_{i - 1}$ is time interval between two measurements.

It is possible to achieve the required accuracy of estimating the angular velocity, taking into account the measuring error of the magnetometers themselves^17^.

## Reconstruction of the disturbing effect on the small spacecraft prototype "Aist"

**Figure 6:**
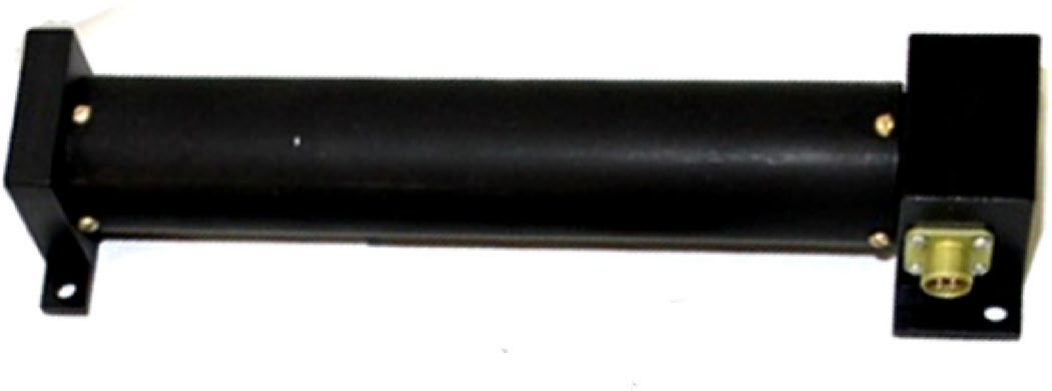
There is one of three electromagnets installed on the small spacecraft prototype "Aist". (cited in^4^).

In general, the small spacecraft prototype "Aist" was operated in uncontrolled flight^3,6^. However, three attempts were made to reduce the angular velocity of its rotation (Fig. 2) using magnetic actuators (Fig. 6). Its main characteristics are shown in Table 2.

**Table 2:** Main characteristics of magnetic actuators.

№	Name	Dimension	Value
1	Working voltage	*V*	5
2	Maximum allowable current	*A*	0,7
3	Maximum magnetic moment on each channel	*A*·*m*^2^	± 0,5
4	Residual magnetic moment on each channel	*A*·*m*^2^	± 0,005

When carrying out a numerical analysis, we will introduce the following assumptions.

- The model of the Earth's magnetic field is described by the International Geomagnetic Reference Field (IGRF) (for example^6,15^).
- The measuring instruments operated correctly, and the differences significance between them is explained solely by the influence of the target equipment operation.
- The components measurements of the Earth's magnetic field were carried out so often that the total time derivative $\dot{\vec{B}}$ of the magnetic induction vector in formula (11) is negligible compared to the local derivative $\frac{{\tilde{d}\vec{B}}}{d\,t}$.
- The components measurements of the Earth's magnetic field were carried out with a uniform step and a frequency sufficient for the correct application of the Kotelnikov theorem when restoring a continuous signal from discrete readings.

We will consider three different situations when reconstructing the disturbance.

1) *The shadow section of the orbit near the magnetic equator with the electromagnets turned off.*

This site is characterized by:

- the absence of the battery charging current in the on-board network of a small spacecraft (Fig. 4);
- low values of the modulus of the induction vector of the Earth's magnetic field;
- low values of the magnetic moment of a small spacecraft;
- the lack of a control torque of the magnetic actuators.

**Figure 7:**
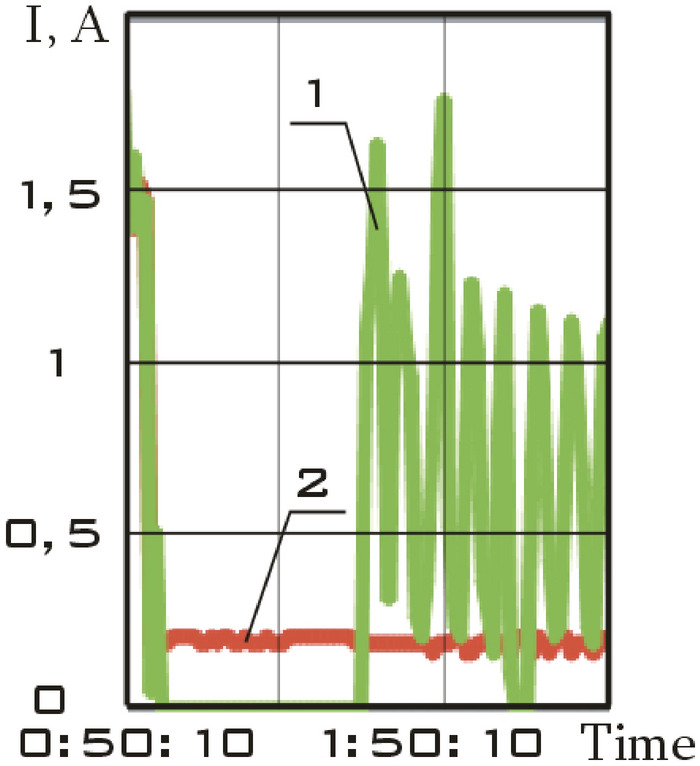
There is dynamics of currents in the power supply system of the small spacecraft prototype "Aist" according to telemetry data from 01/11/2014: 1 is battery charging current; 2 is current of power consumption of scientific equipment.

The segment from 01/11/2014 from 01:45:14 to 01:48:20 was selected as such a section. Current data is shown in Fig. 7.

This area is characterized not only by a zero charging current of the storage battery, but also by low values of the current of power consumption of scientific equipment (Fig. 7). Therefore, the magnetic disturbing moment for this section should be less in magnitude with a correct assessment than for other sections.

Let us estimate, from the measurement data, the values of the components of the angular velocity vector in the *CXYZ* coordinate system using formulas (13). We will carry out their joint processing since there were two magnetometers on board the small spacecraft prototype "Aist". We will take into account the following circumstances when assigning weights. It is noted in^6^ that the measurements of magnetometer No. 2 are noticeably more accurate than measurements of magnetometer No. 1. Therefore, the authors of^6^ did not take into account the measurements of magnetometer No. 1 when reconstructing the rotational motion. It is indicated in^18,19^ that the measurement channel of magnetometer No. 1 [X_1_] differs significantly from the related channel of measurements of magnetometer No. 2 [Y_2_]. In this case, channel [X_1_] stands out among all measurement channels in that, apart from the areas of operation of the magnetic actuators, its mean value and sample variance are the smallest. The anomalousness of the channel [X_1_] was checked in^18^. However, ignorance of the distribution law of the measured value forced us to restrict ourselves to the use of nonparametric criteria. It was not possible to recognize this measurement channel as an outlier as a result of checking with several criteria,. It should be understood that inaccuracies in the measurements of magnetometer No. 1 are most likely associated not with its incorrect operation, but with the influence of the target and supporting equipment^19^. Therefore, in this work, a joint processing of measurements is carried out in contrast to^6^. However, doubts about the measurements of the magnetometer No. 1 led to the appointment of the weighting coefficients $\delta_{1} = 0,2$ and $\delta_{2} = 0,8$. It is this ratio that gives the value of the modulus of the magnetic induction vector, which is the closest to the reference field of the IGRF.

The point estimates of the components of the angular velocity vector in the *CXYZ* coordinate system obtained as a result of joint processing were restored to continuous dependences using the Kotelnikov series^20^:

14 $$\omega_{k} \left( t \right) = \sum\limits_{i = 0}^{n} {\frac{{\left( { - 1} \right)^{n} \omega_{ki} }}{t - n\Delta \,t}} \frac{{\sin \left( {\frac{\pi \,t}{{\Delta \,t}}} \right)}}{{\frac{\pi \,t}{{\Delta \,t}}}}$$

where $\Delta \,t = 6\,\,s$ is uniform interval between measurements; $k = x,y,z$.

**Figure 8:**
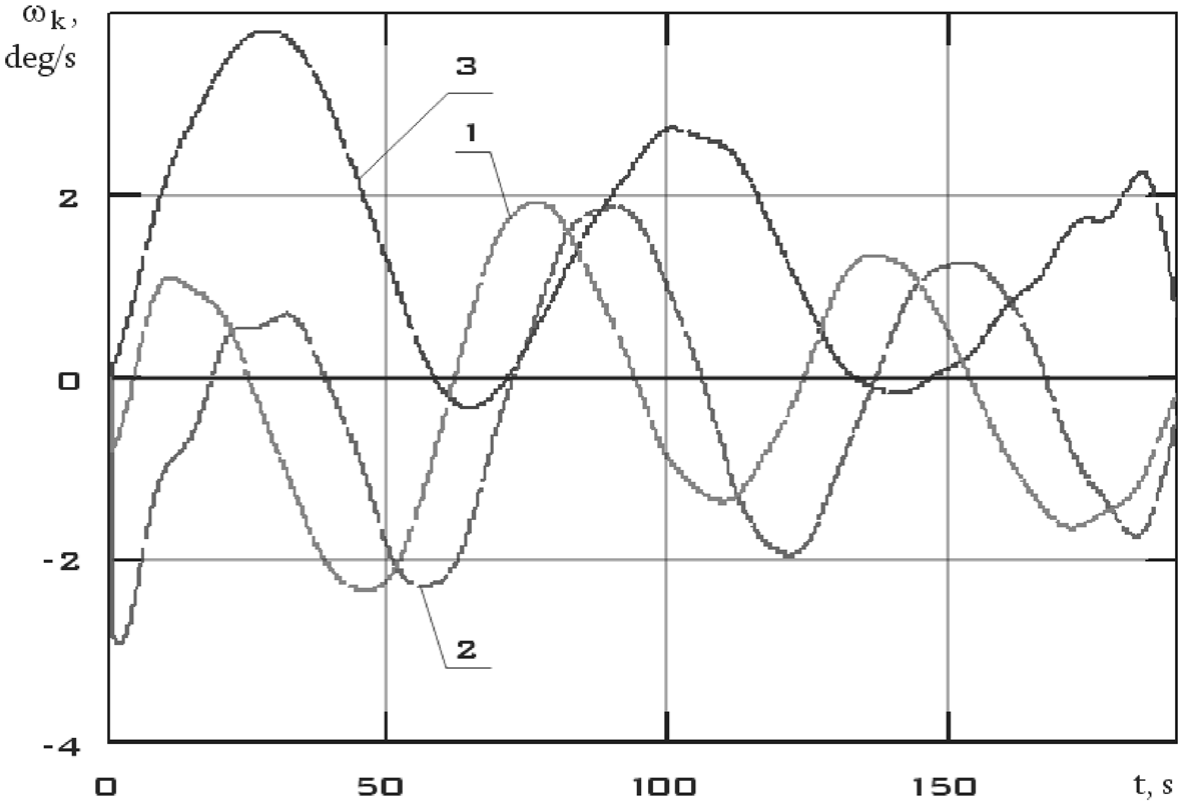
There are components of the angular velocity vector in the *CXYZ* coordinate system. 01/11/2014 from 01:45:14 to 01:48:20 (1 is $\omega_{x}$; 2 is $\omega_{y}$; 3 is $\omega_{z}$).

The results of recovery by formula (14) are shown in Fig. 8.

**Figure 9:**
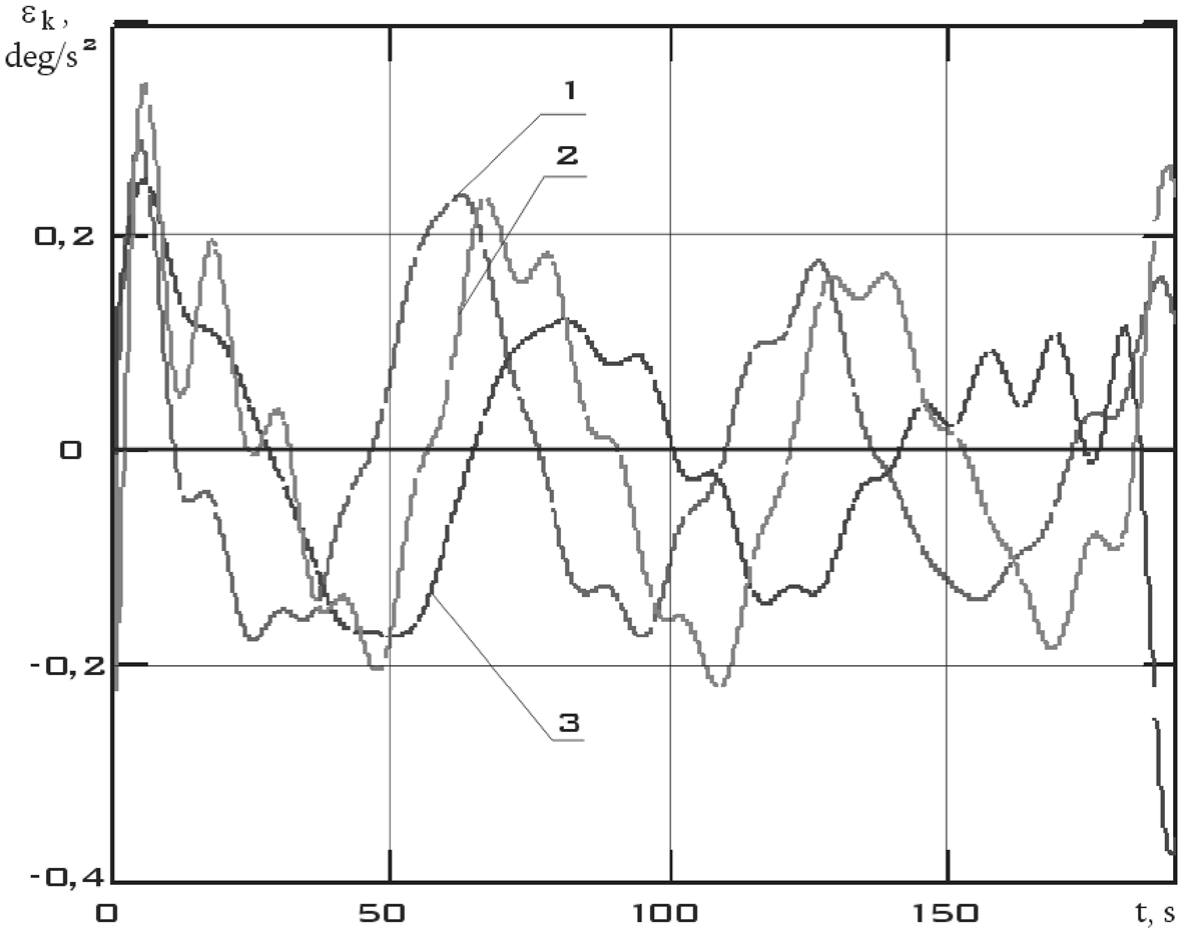
There are components of the angular acceleration vector in the *CXYZ* coordinate system 01/11/2014 from 01:45:14 to 01:48:20 (1 is $\varepsilon_{x}$; 2 is $\varepsilon_{y}$; 3 is $\varepsilon_{z}$).

The components of the angular acceleration vector of the small spacecraft prototype "Aist" were obtained after obtaining continuous dependences of the components of the angular velocity vector by time differentiation (Fig. 9).

**Figure 10:**
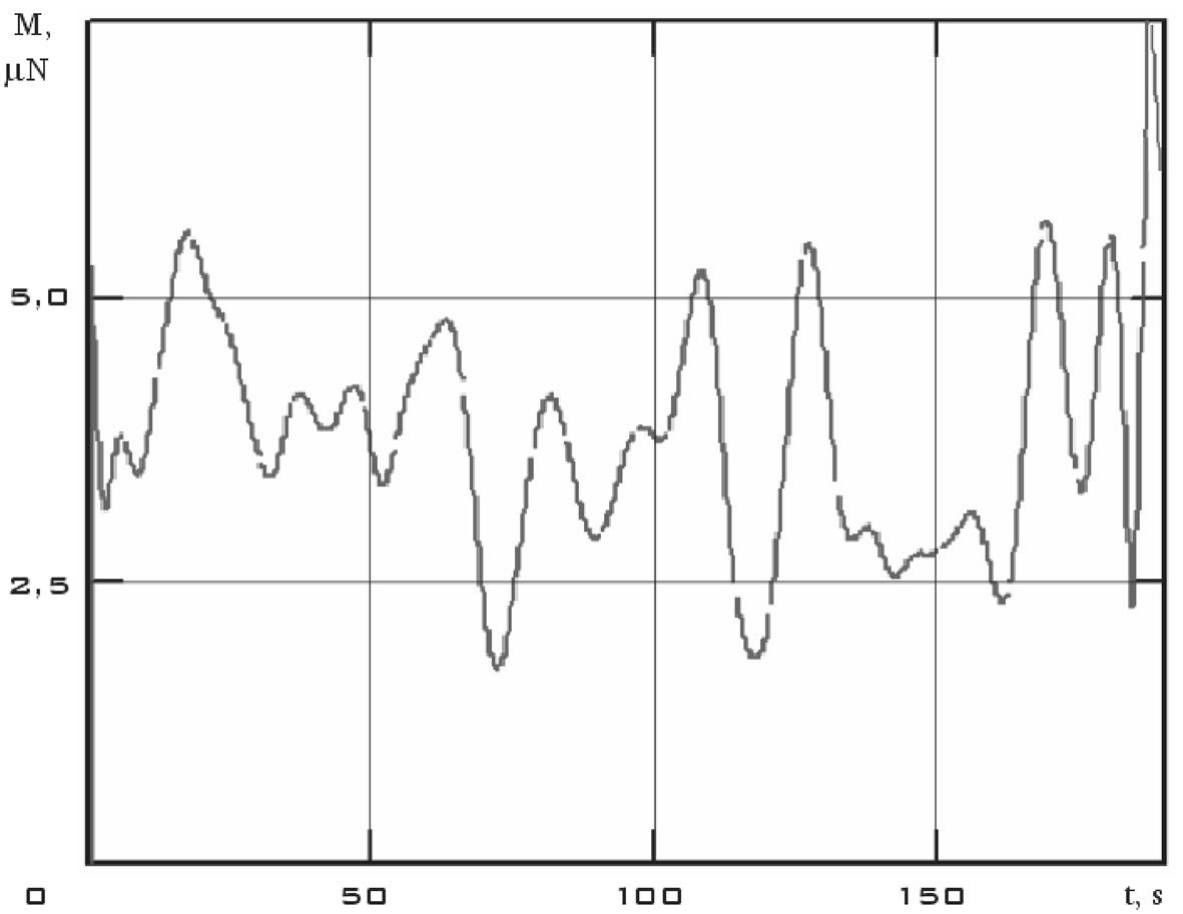
There are modulus of the disturbing moment in the coordinate system *CXYZ.* 01/11/2014 from 01:45:14 to 01:48:20.

The components of the external disturbing moment were estimated and the dependence of its modulus on time in the considered time interval was obtained from the measurement data using the dynamic Euler Eqs. (1) (Fig. 10).

Analysis of Fig. 10 shows that the results obtained correspond to the assessment (7). It should also be noted that for the small spacecraft prototype "Aist" in the study of its rotational motion around the center of mass, the magnetic disturbance is dominant.

2) *Illuminated section of the orbit near the magnetic pole with the electromagnets turned off.*

This site is characterized by:

- the presence of the battery charging current in the on-board network of a small spacecraft (Fig. 4);
- high values of the modulus of the induction vector of the Earth's magnetic field;
- high values of the magnetic moment of a small spacecraft;
- the lack of a control torque of the magnetic actuators.

**Figure 11:**
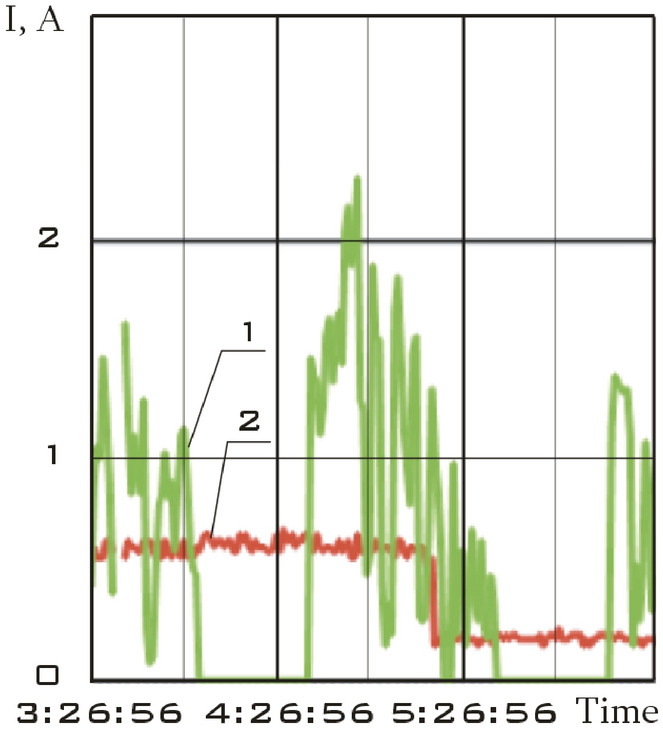
There is dynamics of currents in the power supply system of the small spacecraft prototype "Aist" according to telemetry data from 01/14/2014: 1 is battery charging current; 2 is current of power consumption of scientific equipment.

The segment from 01/14/2014 from 04:41:15 to 04:44:21 was selected as such a section. Current data is shown in Fig. 11.

**Figure 12:**
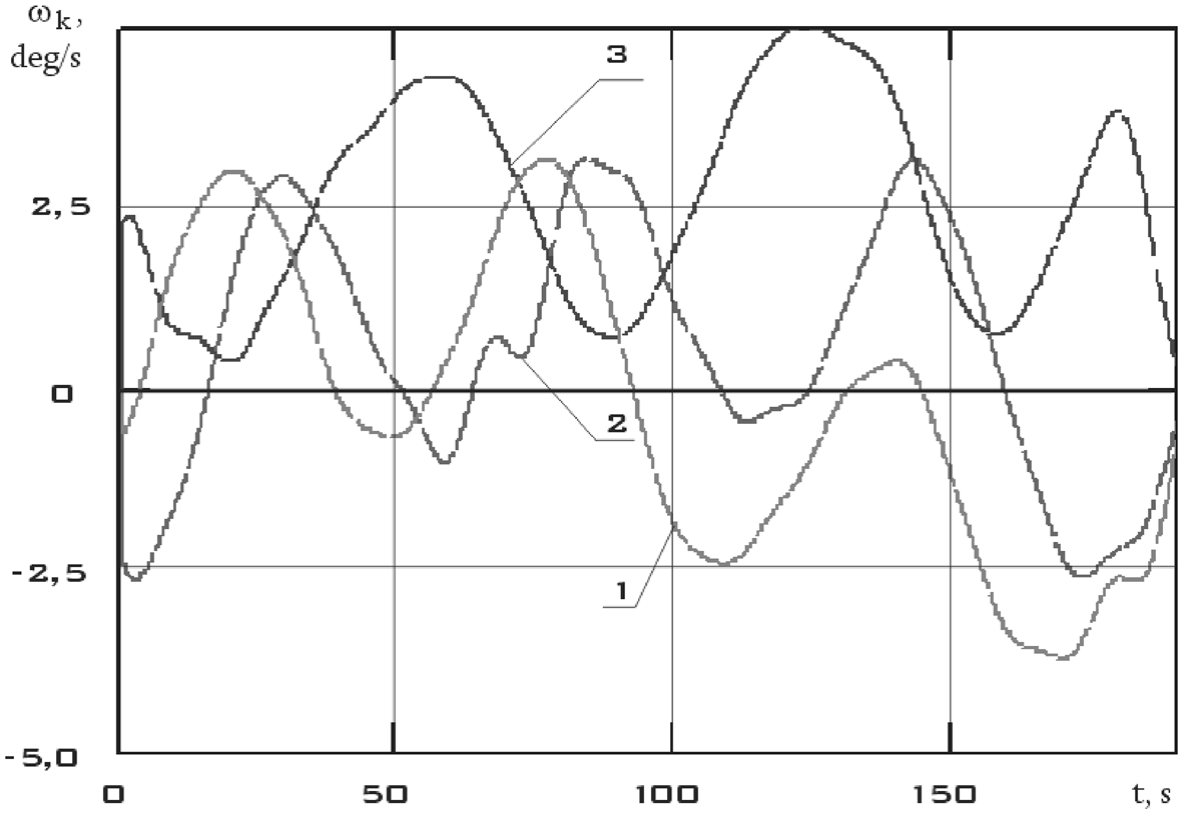
There are components of the angular velocity vector in the *CXYZ* coordinate system. 01/14/2014 from 04:41:15 to 04:44:21 (1 is $\omega_{x}$; 2 is $\omega_{y}$; 3 is $\omega_{z}$).

**Figure 13:**
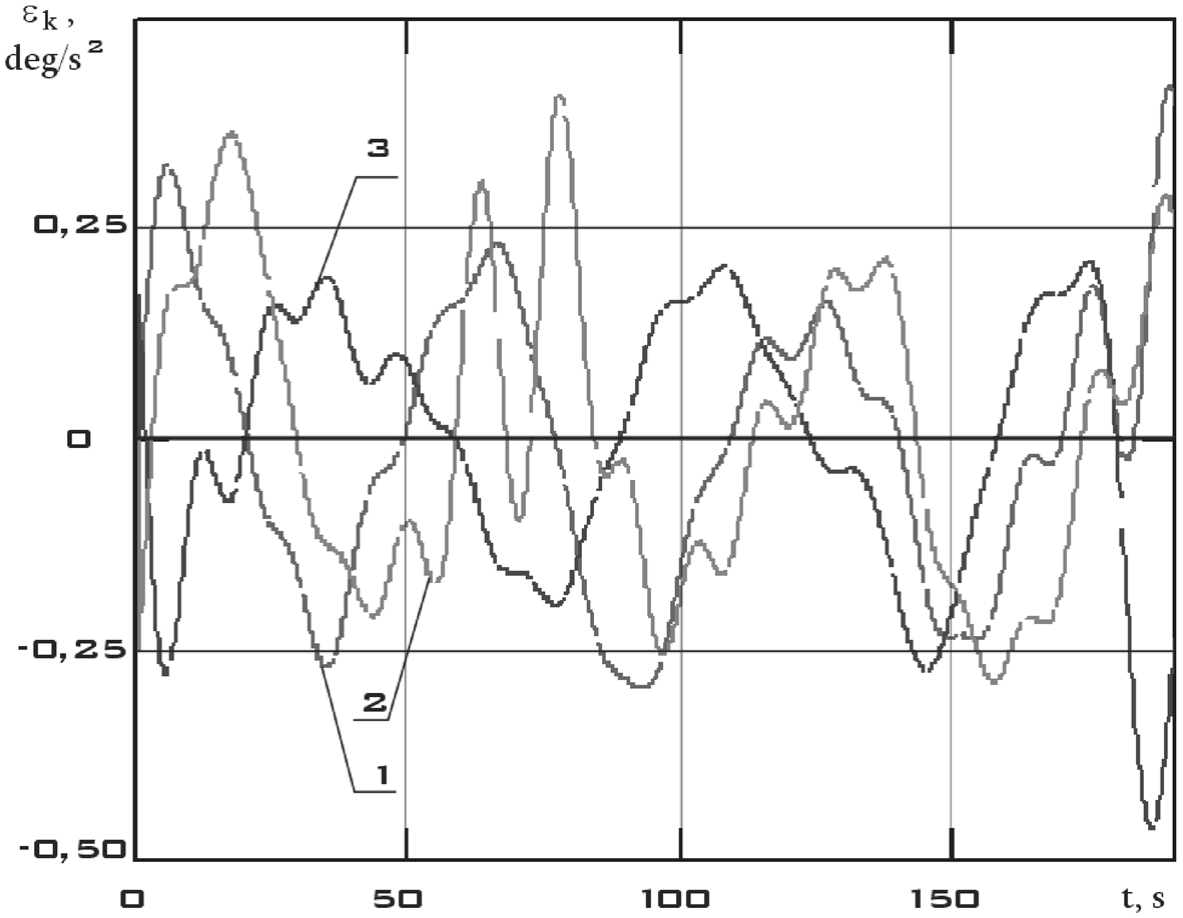
There are components of the angular acceleration vector in the *CXYZ* coordinate system 01/14/2014 from 04:41:15 to 04:44:21 (1 is $\varepsilon_{x}$; 2 is $\varepsilon_{y}$; 3 is $\varepsilon_{z}$).

This section is characterized not only by high values of the battery charging current, but also by high values of the energy consumption current of scientific equipment unlike the previous one (Fig. 11). Thus, a significant increase in the disturbing moment should be observed with the correctness of the estimation of the disturbing moment from the measurement data and the adequacy of the assumption about the dominance of magnetic disturbances. The continuous dependences of the components of the angular velocity vector of a small spacecraft in the selected area, reconstructed by the Kotelnikov series (14), are shown in Fig. 12. The corresponding components of the angular acceleration vector are shown in Fig. 13.

**Figure 14:**
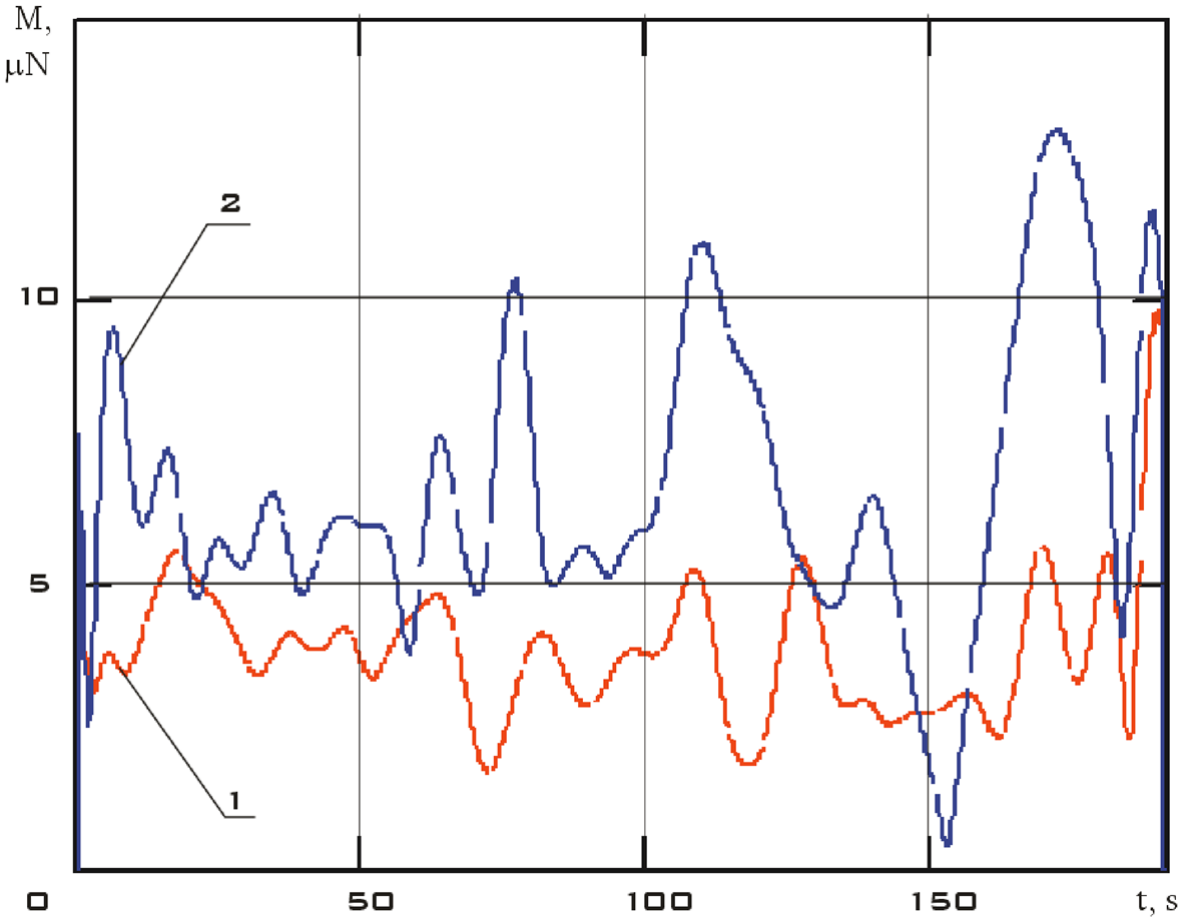
There are modulus of the disturbing moment in the coordinate system *CXYZ*. 1—01/11/2014 from 01:45:14 to 01:48:20; 2—01/14/2014 from 04:41:15 to 04:44:21.

It can be seen that the angular acceleration in the second section is noticeably higher comparing Figs. 9 and 11. It was to be expected in the case of the correctness of the estimate of the force disturbances. The modulus of the disturbing moment acting on the small spacecraft prototype "Aist" in the area under consideration is shown in Fig. 14.

Figure 14 shows the modulus of the disturbing moment from Fig. 10 for clarity of comparison. It can be seen that curve 2 as a whole is noticeably higher than curve 1. The same picture is observed in the comparison of other illuminated and shadow parts of the orbit. Thus, it can be argued that the qualitative picture of the restoration of the disturbing effect looks quite realistic. Let's check the quantitative correspondence. We will select measurement areas with working magnetic actuators for this purpose. The magnetic actuators were used only three times according to Fig. 2. Therefore, the selection of areas is not as wide as in the case of switched off magnetic actuators.

3) *Orbital section with electromagnets turned on.*

This site is characterized by:

- the presence of a control torque of the magnetic actuators;
- the availability of information about the strength of the current supplied to the magnetic actuators.

**Figure 15:**
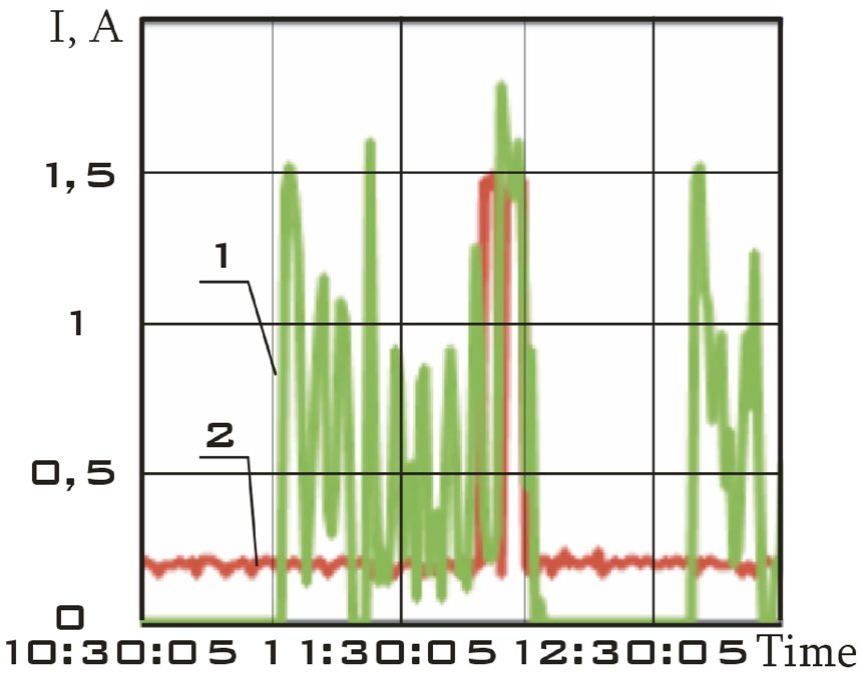
There is dynamics of currents in the power supply system of the small spacecraft prototype "Aist" according to telemetry data from 02/15/2014: 1 is battery charging current; 2 is current of power consumption of scientific equipment.

Let us choose as such a section the segment from 02/15/2014 from 12:00:06 to 12:02:21. Current data is shown in Fig. 15.

**Figure 16:**
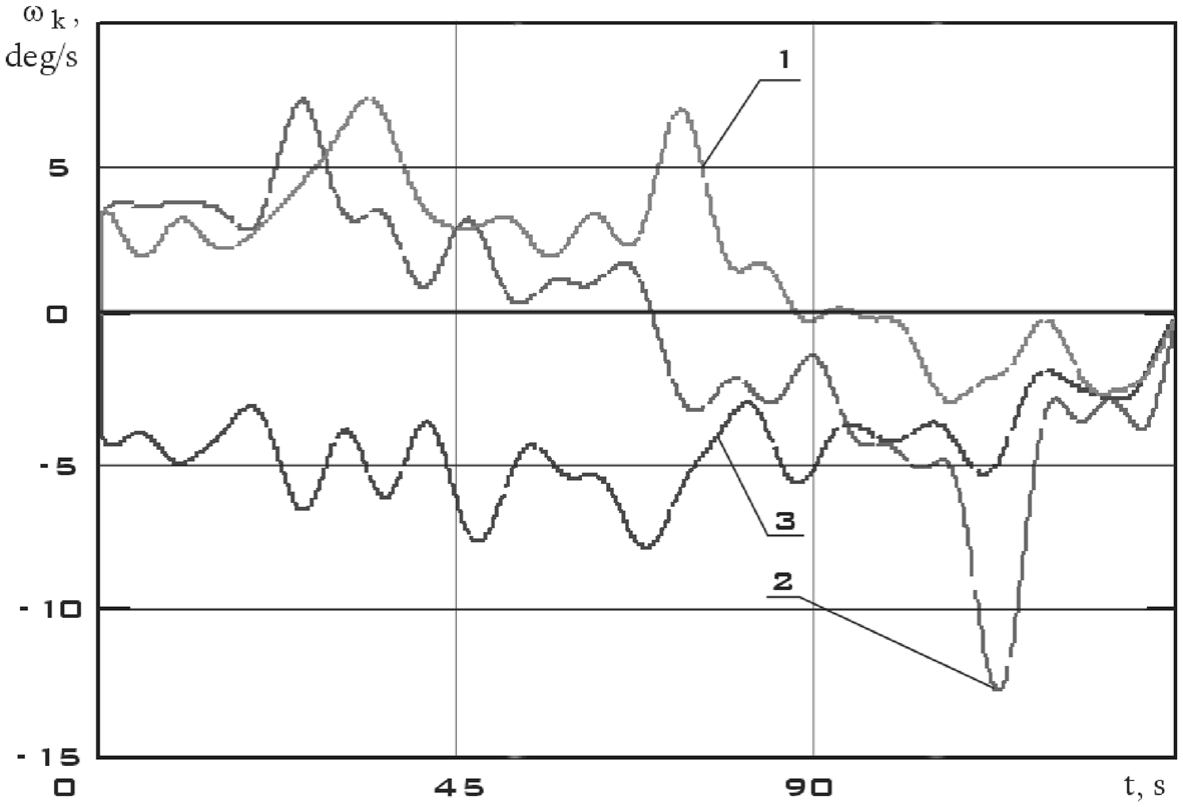
There are components of the angular velocity vector in the *CXYZ* coordinate system. 02/15/2014 from 12:00:06 to 12:02:21 (1 is $\omega_{x}$; 2 is $\omega_{y}$; 3 is $\omega_{z}$).

**Figure 17:**
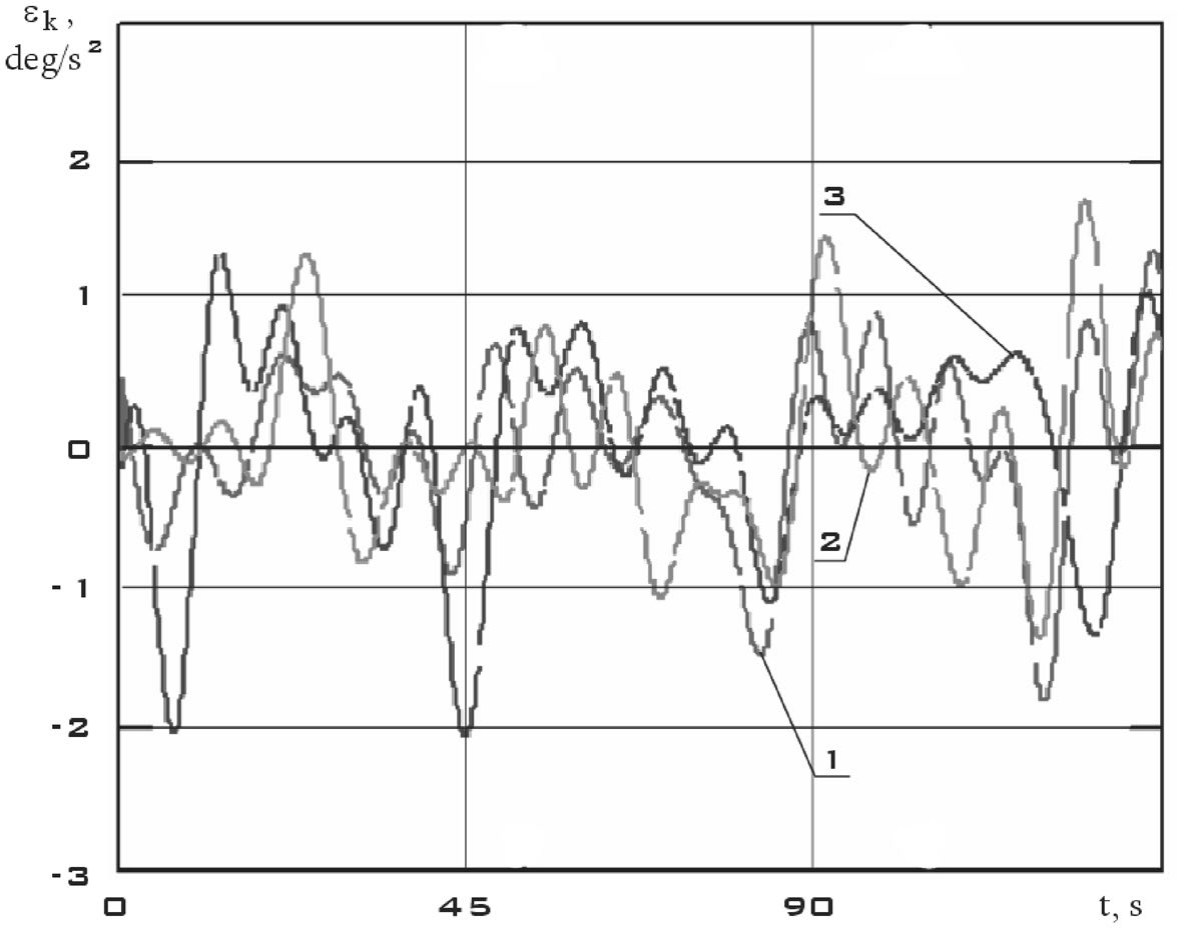
There are components of the angular acceleration vector in the *CXYZ* coordinate system 02/15/2014 from 12:00:06 to 12:02:21 (1 is $\varepsilon_{x}$; 2 is $\varepsilon_{y}$; 3 is $\varepsilon_{z}$).

The dependencies of the components of the angular velocity and angular acceleration vectors of a small spacecraft in the selected area are shown in Figs. 16 and 17, respectively.

**Figure 18:**
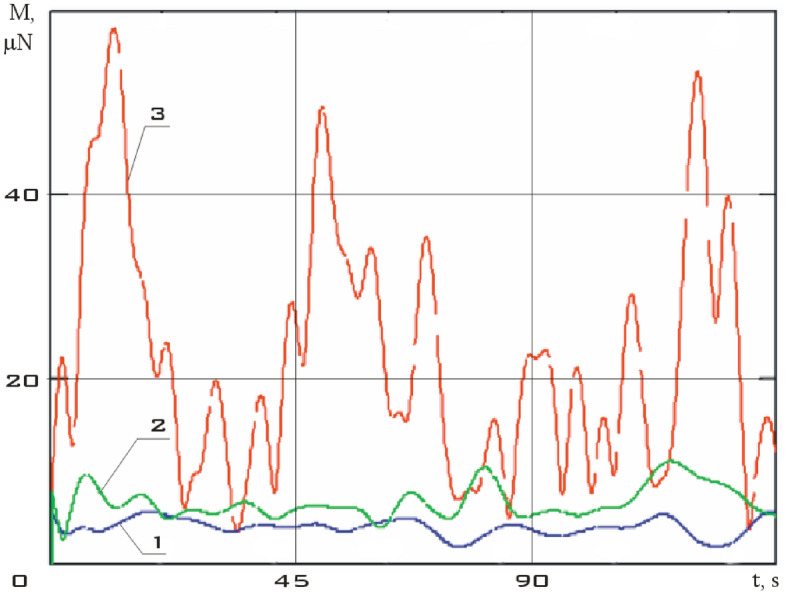
There are modulus of the disturbing moment in the coordinate system *CXYZ*. 1 is 01/11/2014 from 01:45:14 to 01:48:20; 2 is 01/14/2014 from 04:41:15 to 04:44:21; 3 is 02/15/2014 from 12:00:06 to 12:02:21.

An estimate of the modulus of the disturbing moment was obtained using estimates of the angular velocity and angular acceleration, as well as model (1). It is presented in Fig. 18 in comparison with previous estimates (Fig. 14).

The disturbing moment during the operation of the magnetic actuators is more than 5 times higher than the disturbing moment when the magnetic actuators are turned off as can be seen from Fig. 18. This conclusion corresponds to the real picture.

**Figure 19:**
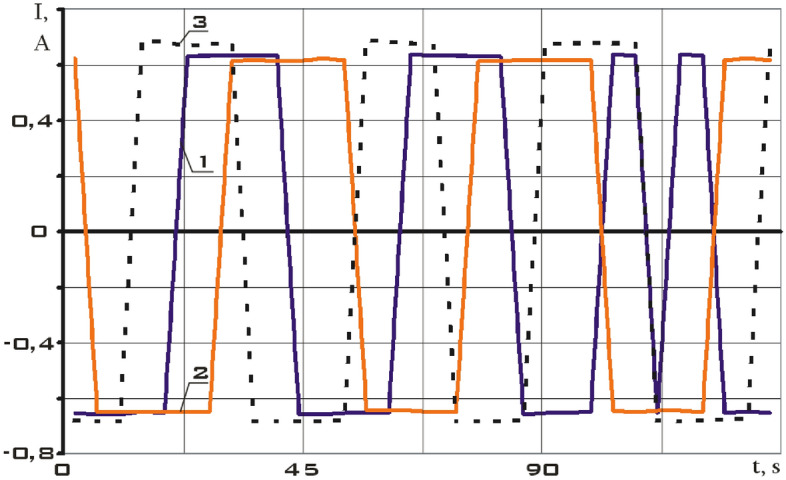
There is the current supplied to the magnetic actuators of the small spacecraft prototype "Aist" according to telemetry data from 02/15/2014 from 12:00:06 to 12:02:21. 1 is on an electromagnet, the axis of which is parallel to the *CX* axis; 2 is on an electromagnet, the axis of which is parallel to the *CY* axis; 3 is on an electromagnet, the axis of which is parallel to the *CZ* axis.

Let us estimate the magnetic moment of interaction of the actuators of the small spacecraft prototype "Aist" with the Earth's magnetic field according to the data on the current strength supplied to the actuators and the data of the components measurements of the induction vector of the Earth's magnetic field. The data on the current strength are presented in Fig. 19 on the segment 02/15/2014 from 12:00:06 to 12:02:21.

**Figure 20:**
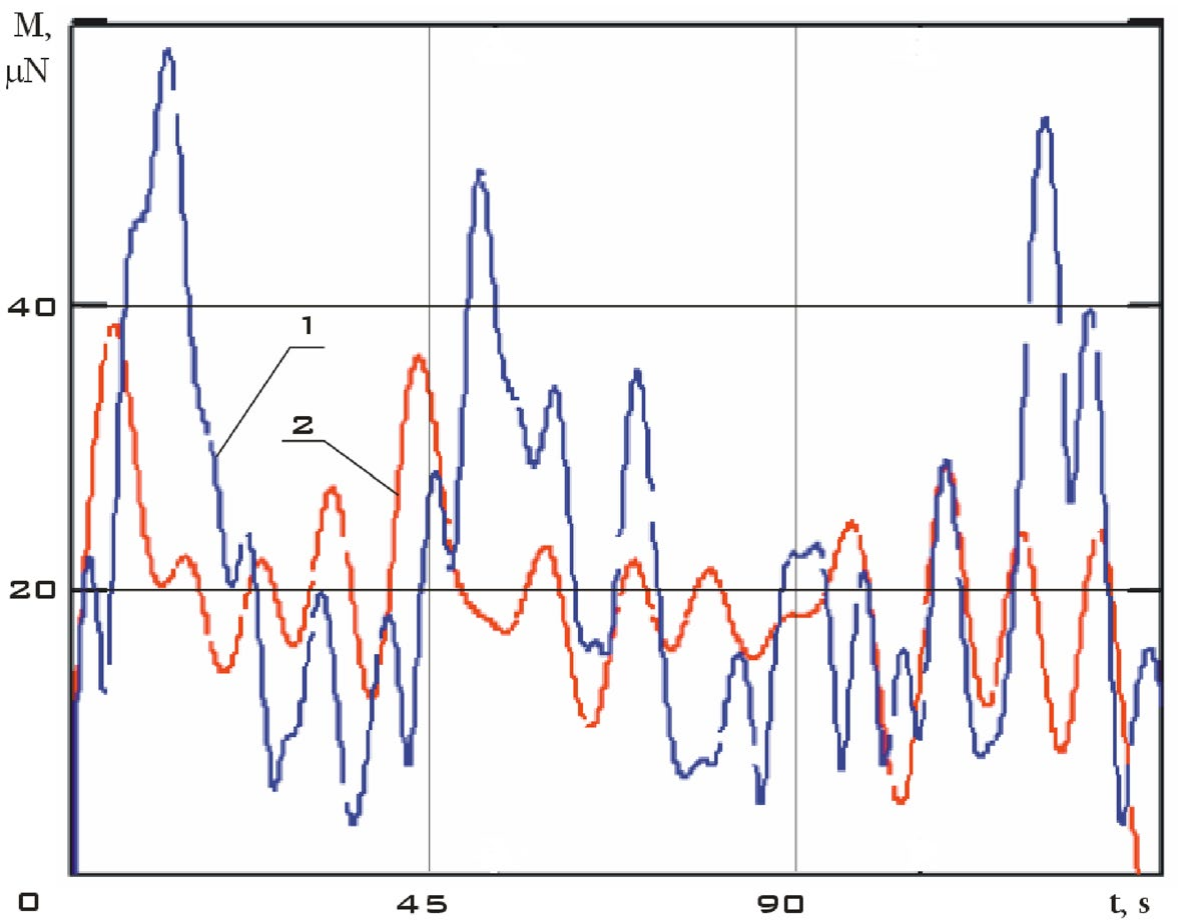
There are modulus of the disturbing moment in the *CXYZ* coordinate system. 1 is according to model (1) taking into account all disturbances; 2 is according to the formula (7), taking into account the magnetic interaction of the actuators with the Earth's magnetic field.

It is necessary to restore the modulus of the moment by the Kotelnikov series (14) for a correct comparison of the moduli of the disturbing moments obtained using model (1) and formula (7). The results are shown in Fig. 20.

Let us carry out a comparative analysis of the curves shown in Fig. 20. The data on the angular velocity of rotation of a small spacecraft, estimated by formulas (13), are used when calculating the disturbing moment according to model (1). Consequently, all disturbances, including those of a non-magnetic nature, are taken into account here. Formula (7) estimates only the magnetic moment of interaction of the actuators with the Earth's magnetic field. In fact, the disturbances shown by curve 2 in Fig. 20 are part of the general disturbances and are included in the disturbances shown by curve 1. It explains the differences in the amplitudes of curves 1 and 2.

The change in the composition of the disturbing factors can also explain the stabilization of the small spacecraft, shown in Fig. 2. As the battery degrades, the scientific and supporting equipment ceases to function. In this case, the small spacecraft turns into an object with a constant dipole moment. And this object, according to known laws, is stabilized in the Earth's magnetic field^21^.

On the other hand, magnetic disturbances not related to the work of the actuators are still significant. It is their neglect that largely affects the difference between curves 1 and 2. Differences within the maximum values of curve 10 can be explained by magnetic disturbances not related to the operation of the actuators.

The issue of taking into account perturbations of a non-magnetic nature should be attributed to a specific problem to be solved. So, these perturbations can be ignored to assess microaccelerations, based on the data in Table 1. Table 1 shows the maximum values of disturbances that might not have been included in the above examples. So, for example, the maximum value of the moment from the magnetic actuators, according to formula (7), will be observed near the poles. Since the modulus of the induction vector of the Earth's magnetic field is maximum there. However, control near the poles is not effective due to significant changes in the direction of the magnetic induction vector, even in a small part of the orbit.

The errors estimation of the presented results can be carried out taking into account the results of works^17^ and^21^. In^17^, the measuring error is estimated for the measuring instruments themselves. In^21^, the influence of the operation of the target and supporting equipment on the measurement error is taken into account. At the same time, the decrease in the measurement accuracy due to this influence is estimated. Quantitative estimates of these errors at the level of measuring instruments were discussed in^18^. Their maximum values are approximately $\pm 7\,\,\mu T$ for the peak load of scientific equipment and $\pm 10\,\,\mu T$ for the operation of magnetic actuators and scientific equipment. Such high values are explained by the super-dense arrangement of the equipment inside the small spacecraft. Its total volume is only 0.13 *m*^2^^18^.

## Conclusion

Thus, the work carried out the reconstruction of the disturbances acting on the small spacecraft prototype "Aist". The solar and shadow parts of the orbit with the magnetic actuators turned off, as well as the area with working magnetic actuators are analyzed. The dominant influence of magnetic disturbances on the rotational motion of the small spacecraft prototype "Aist" was revealed in all three areas. It was found that in the shadow part of the orbit, the disturbances are noticeably lower than in the solar part. It is due to the absence of the battery charging current and, as a consequence, the decrease in the magnetic moment of the small spacecraft. The perturbations of a magnetic nature increase significantly during the operation of magnetic actuators. However, it is apparently incorrect to consider only the magnetic moment of interaction between the actuators and the Earth's magnetic field. Magnetic disturbances from the operation of the target and support equipment are still significant. They must be taken into account when evaluating microaccelerations, otherwise this estimate may be significantly underestimated.
